# Retinitis Albuminuria

**Published:** 1877-10-20

**Authors:** 


Retinitis Albuminurica.—By George C. Harlam, M. D., Surgeon to Will's Opthalmic Hospital, Philadelphia; also, Hemiopia and Decussation in the Optic Chiasm, by the same author.

